# Supplementary material for: Time‑resolved multi-omic analysis of paclitaxel exposure in human iPSC‑derived sensory neurons unveils mechanisms of chemotherapy‑induced peripheral neuropathy
Source: Cell Death Dis. 2026 Feb 10;17(1):211. doi: 10.1038/s41419-026-08445-2 (PMC12921266; doi:10.1038/s41419-026-08445-2)
Supplement: Supplementary file 15 — Dataset 1. [file 41419_2026_8445_MOESM15_ESM.zip › CDD_Open_Data/04_RNA_Seq/Timeline_panel_plots_etc_2023-01-23_1609.zip!/Panel_Plots_etc/Evidence_Plots/post5d/evidence_plot_standard contrasts_msigdb_reactome_5d_PTX_vs_DMSO_ID6_M27172_REACTOME_ACTIVATION_OF_GENE_EXPRESSION_BY_SREBF_SREBP.pdf]

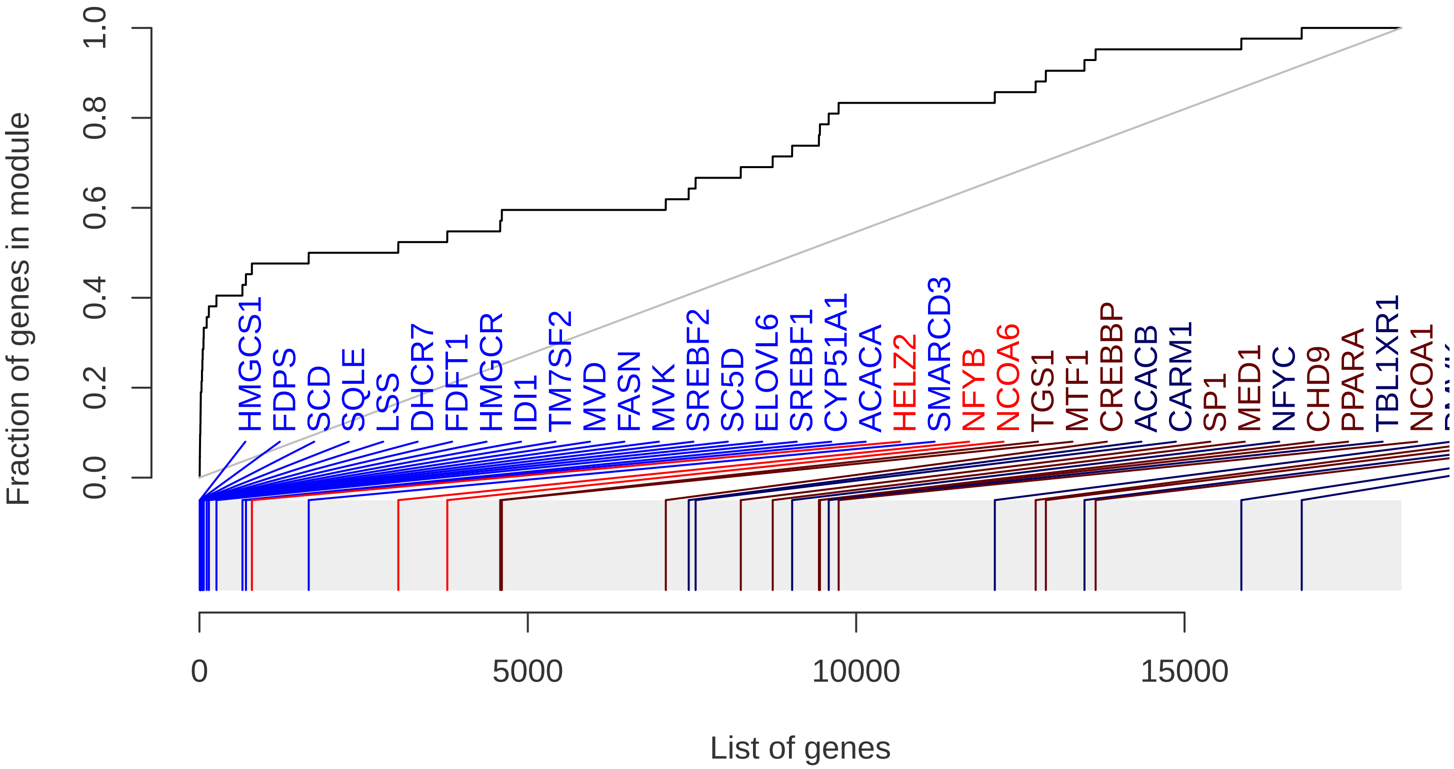

Fraction of genes in module
0.0
0.2
0.4
0.6
0.8
1.0
List of genes
0
5000
10000
15000
HMGCS1
FDPS
SCD
SQLE
LSS
DHCR7
FDFT1
HMGCR
IDI1
TM7SF2
MVD
FASN
MVK
SREBF2
SC5D
ELOVL6
SREBF1
CYP51A1
ACACA
HELZ2
SMARCD3
NFYB
NCOA6
TGS1
MTF1
CREBBP
ACACB
CARM1
SP1
MED1
NFYC
CHD9
PPARA
TBL1XR1
NCOA1
